# Identification of the Position of a Tethered Delivery Catheter to Retrieve an Untethered Magnetic Robot in a Vascular Environment

**DOI:** 10.3390/mi14040724

**Published:** 2023-03-24

**Authors:** Serim Lee, Nahyun Kim, Junhyoung Kwon, Gunhee Jang

**Affiliations:** Department of Mechanical Convergence Engineering, Hanyang University, Seoul 04763, Republic of Korea; tpfla8243@hanyang.ac.kr (S.L.); shqpffhtm@hanyang.ac.kr (J.K.)

**Keywords:** position identification, retrieval of an untethered magnetic robot, vascular environment

## Abstract

In this paper, we propose a method of identifying the position of a tethered delivery catheter in a vascular environment, recombining an untethered magnetic robot (UMR) to the tethered delivery catheter, and safely retrieving them from the vascular environment in an endovascular intervention by utilizing a separable and recombinable magnetic robot (SRMR) and a magnetic navigation system (MNS). From images of a blood vessel and a tethered delivery catheter taken from two different angles, we developed a method of extracting the position of the delivery catheter in the blood vessel by introducing dimensionless cross-sectional coordinates. Then, we propose a retrieval method for the UMR by using the magnetic force considering the delivery catheter’s position, suction force, and rotating magnetic field. We used thane MNS and feeding robot to simultaneously apply magnetic force and suction force to the UMR. In this process, we determined a current solution for generating magnetic force by using a linear optimization method. Finally, we conducted in vitro and in vivo experiments to verify the proposed method. In the in vitro experiment, which was in a glass tube environment, by using an RGB camera, we confirmed that the location of the delivery catheter in the glass tube could be recognized within an average error of 0.05 mm in each of the X- and Z-coordinates and that the retrieval success rate was greatly improved in comparison with that in the case without the use of magnetic force. In an in vivo experiment, we successfully retrieved the UMR in the femoral arteries of pigs.

## 1. Introduction

Occlusive vascular diseases (OVDs), such as stroke in the brain, myocardial infarction in the heart, and peripheral artery disease in the leg, occur when a blood vessel becomes narrowed or blocked due to accumulated blood clots and lipids. Such diseases are expected to become even more important health issues globally, since the incidence rate of OVDs is certain to increase due to bad eating habits and an aging society [1]. OVDs are treated through endovascular interventions, in which medical doctors first secure a path through existing blood vessels to blocked lesions by using catheters and guidewires, and then insert a balloon or a stent through the secured path to widen the lesion [2,3]. During the procedure, medical doctors monitor the blocked lesion, guidewires, and catheters through *X*-ray imaging devices. This procedure has become popular because this treatment does not entail the opening of the body. However, since medical doctors can manipulate only the proximal part of a conventional tethered device, which is located outside of the patient’s body, it is almost impossible to precisely control the magnitude and direction of the force and the torque at the distal end of the device [4,5,6]. Furthermore, medical doctors are continually exposed to hazardous *X*-ray radiation [7,8,9]. As an alternative for overcoming these limitations, a magnetic robot system has been proposed [10].

The magnetic robot system comprises a magnetic navigation system (MNS) that generates an external magnetic field and a magnetic robot to which magnetic materials are attached. A magnetic robot in a blood vessel can be controlled with the magnetic torque or force that results from the interaction of the external magnetic field and the magnetic materials [11,12]. Through the remote control of the magnetic robot with an external magnetic system, the medical doctors can be protected from exposure to *X*-ray radiation. By precisely controlling the strength and direction of the magnetic field, it is possible to actively control the magnetic robot and perform various treatment functions, such as drug delivery and tunneling of the lesion. These magnetic robots are primarily classified as tethered or untethered magnetic robots according to their mechanical structure.

Tethered magnetic robots are virtually a catheter and guidewire with a permanent magnet at the distal part, and they can be steered with an external magnetic field and perform various treatment functions. Many studies have been conducted on precise steering to a target position and on treatment mechanisms [12]. Lee et al. proposed a structure of a tethered magnetic robot that was capable of remotely performing various treatment functions, such as steering, tunneling, and stent delivery [13]. In addition, Jeon et al. improved the steerability of a magnetic catheter with two permanent magnets in comparison with conventional guidewires [14]. Tethered magnetic robots have the advantage of being immediately retrieved outside the body at any instant before and after performing various treatment functions. However, in the case of blood vessels with complex shapes, buckling may occur along the tethered magnetic robot, which makes it difficult to advance any further because the force applied to the proximal part is not efficiently transmitted to the distal end [11]. On the other hand, an untethered magnetic robot (UMR) with a helical shape was proposed to overcome the limitations of tethered magnetic robots. A UMR can be propelled by magnetic torque that is generated by the interaction of the permanent magnet of the UMR and the rotating magnetic field from the MNS [15,16,17,18]. Many researchers have investigated UMRs, mainly in terms of their driving and tunneling performance. However, it is inefficient to insert a UMR into a superficial artery to reach a target lesion—which is usually located deep in the body—for a short time because it is not easy to precisely steer the robot through several bifurcated blood vessels and to circumvent the fast blood flow of the arteries. Several researchers proposed a separable and recombinable magnetic robot (SRMR) in which a UMR was connected to a tethered magnetic robot [17,18]. An SRMR can efficiently reach the vicinity of a target lesion with combined tethered and untethered structures. Then, the UMR can be separated at the target lesion, perform tunneling, and be recombined with the tethered magnetic robot. Park et al. proposed an SRMR that combined a UMR with a permanent magnet at the end of a tethered magnetic robot [17]. However, a magnetic force in an unintentional direction could be generated during the steering motion, or an external force could separate it during delivery to the lesion. Sa et al. proposed another form of SRMR in which a UMR could be separated and recombined with a delivery catheter by using a screw mechanism and rotating external magnetic field [18]. They proposed a method of recombination of the delivery catheter and UMR by applying magnetic force, suction force, and a rotational magnetic field. They did not consider the location of the delivery catheter in the blood vessel. and they only applied magnetic force in order to cause the UMR to levitate, regardless of the location of the delivery catheter. When the UMR was not aligned with the central axis of the delivery catheter, it could not be combined with the delivery catheter, and the UMR was not retrieved. Repeated failure of the UMR retrieval process delays operation times and can be hazardous to the patients.

In this paper, we propose a method of identifying the position of a tethered delivery catheter in a vascular environment by utilizing images of a blood vessel and tethered delivery catheter taken from two arbitrary angles, aligning a UMR with the tethered delivery catheter by applying magnetic force while considering the delivery catheter’s position, recombining the UMR with the delivery catheter by using suction force and a rotating magnetic field, and safely retrieving them from the vascular environment in an endovascular intervention. In this process, we determined a current solution for generating magnetic force by using a linear optimization method. Finally, we conducted an in vitro experiment in a glass tube and an in vivo experiment in the femoral arteries of pigs to verify the proposed method.

## 2. Position Identification and Control of an SRMR

### 2.1. The Structure of the SRMR and the Manipulation Method

#### 2.1.1. The Structure of the SRMR

**Figure 1:**
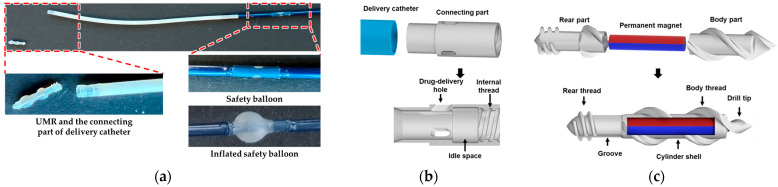
(**a**) An SRMR composed of a tethered delivery catheter with a safety balloon and an untethered UMR; (**b**) structure of the connecting part of the delivery catheter; (**c**) structure of the UMR.

Figure 1a shows the mechanical structure of the SRMR used in this study. It comprised a tethered delivery catheter with a safety balloon and an untethered UMR [18]. During the endovascular intervention, the safety balloon at the distal part of the delivery catheter was inflated to block the blood flow. Figure 1b shows the connecting part of the delivery catheter, and Figure 1c shows the structure of the UMR. The connecting part of the delivery catheter, which had an outer diameter of 2.33 mm, and the rear thread of the UMR, which had an outer diameter of 1.83 mm, were connected by a screw mechanism through the rotational motion of the UMR. The permanent magnet inserted into the UMR was radially magnetized NdFeB (Neodymium-Iron-Boron, N55 grade, Br = 1.47 T) with a length of 5 mm and a diameter of 1 mm, and it was wrapped around the rear part and body part of the UMR. The UMR could perform steering and rotational motions from magnetic torque generated by an external magnetic field and could move upward and downward via a vertical magnetic force. From the rotational motion of the UMR generated by the external rotating magnetic field, the UMR could be axially propelled by pushing the fluid through the helical structure of the body thread, and it could tunnel through thrombin with a drill tip.

#### 2.1.2. A Robotically Adjustable Magnetic Navigation System for Endovascular Interventions (I-RAMAN System)

**Figure 2:**
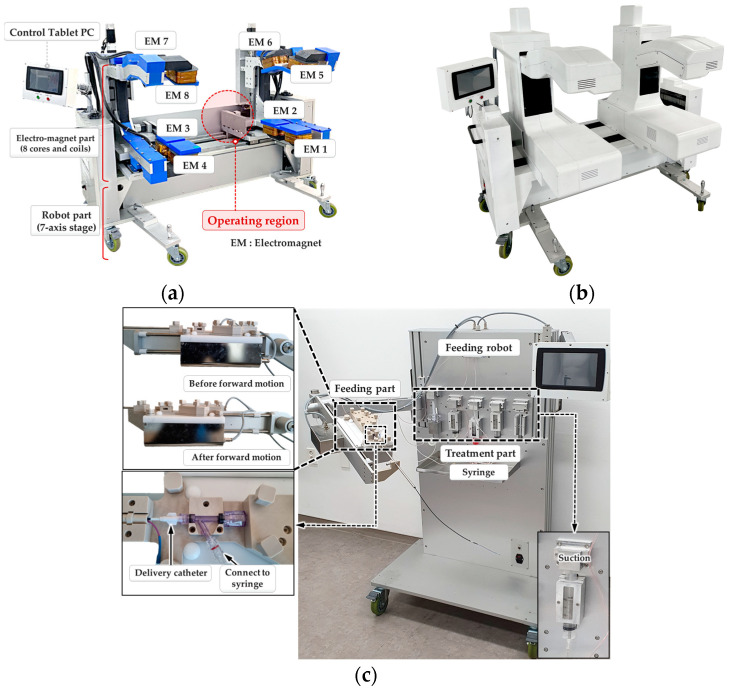
I-RAMAN system: (**a**) structure of the MNS; (**b**) the MNS with exterior housing; (**c**) structure of the feeding robot.

We utilized the I-RAMAN system, which is a robotically adjustable magnetic navigation system for endovascular intervention that comprises a magnetic navigation system (MNS) and a feeding robot for controlling various motions of the UMR and delivery catheter, as shown in Figure 2 [19].

Figure 2a shows the MNS, which consisted of eight electromagnets that were connected by a back yoke. The magnetic flux density generated by an electromagnet is proportional to the applied current. The magnetic field and magnetic force at a point **P**(x,y,z) within an operating region are equal to the superposition of the magnetic fields that are generated by each of the eight electromagnets [20]. The magnetic force applied to the UMR is expressed as **F** = (**m**∙∇)**B,** where **m** is the magnetic moment of the permanent magnet inside the UMR and **B** is the external magnetic flux density from the MNS. Therefore, the relationship between the magnetic flux density **B**(**P**), the magnetic force **F**(**m**,**P**) generated at the point **P**, and the current **I** can be expressed with the following equation by using an actuation matrix $A_{B,F}(m,P)$, which is a 6 × 8 matrix [20].

(1) $$\left[\begin{array}{c} B(P)\\ F(m,P) \end{array}\right]=A_{B,F}(m,P)I$$

The optimal value of the current **I***_opt_* applied to the eight electromagnets to generate the desired magnetic field **B***_des_* and magnetic force **F***_des_* at the target point **P** in the operating region can be expressed with the following equation [19]:

(2) $$\left[\begin{array}{c} B_{des}\\ F_{des} \end{array}\right]=A_{B,F}\left(I_{B}+I_{F}+{c_{1}\cdot }_{null,1}+{c_{2}\cdot I}_{null,2}\right)=A_{B,F}I_{opt}$$

where **I_B_** and **I_F_** are the particular solutions of the current for generating the desired magnetic flux density and magnetic force, respectively, **I_null,1_** and **I_null,2_** are the null spaces of the current, and c_1_ and c_2_ are free variables. The optimal current solution **I***_opt_* can be obtained by applying an optimization method to determine the free variables c_1_ and c_2_ that minimize the norm of the current vector [19]. **I***_opt_* creates the desired magnetic flux density and magnetic force at point **P**.

Meanwhile, the motion of the catheter required to perform therapeutic functions in blood vessels could be precisely controlled outside the patient’s body by using the feeding robot [21,22]. The feeding robot enabled the catheter to move forward and backward and perform suction in the blood vessel. The feeding robot shown in Figure 2c could control the flow speed and rate of suction and injection through the hole of the tube of the delivery catheter by controlling the syringe mounted on the treatment part.

### 2.2. Algorithm for Identifying the Position of the Delivery Catheter in a Vascular Environment

A UMR must be safely retrieved from the patient’s body after the separated UMR performs tunneling in the occluded lesion of a blood vessel. To retrieve a UMR in an endovascular intervention by utilizing an SRMR and MNS, the central axes of the delivery catheter and UMR must be aligned along the length of the blood vessel. The delivery catheter and UMR can be placed close to each other in the blood vessel and can be controlled by the feeding robot and MNS, respectively, and the magnetic force applied to the UMR can be used to align the UMR with the delivery catheter. However, this requires the position information of the delivery catheter and UMR in the cross-section of the blood vessel. In this paper, we assume that the UMR is located at the bottom of the vessel not only because the density of the UMR (5 g/cm^3^) is greater than that of water or blood (1 g/cm^3^), but also because we observed that the UMR was always on the bottom wall of the tube in the experiments.

**Figure 3:**
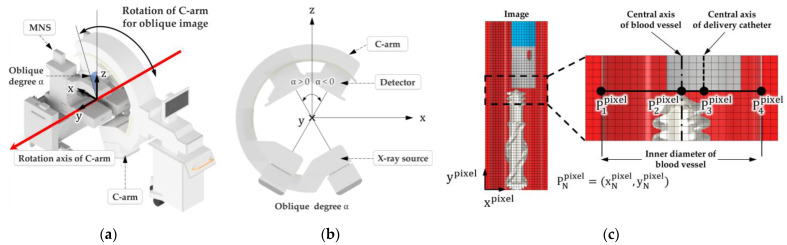
(**a**) Placement of the MNS and C-arm (*X*-ray imaging device) and the rotational axis of the C-arm; (**b**) definition of the oblique angle; (**c**) representation of pixel coordinates in an image.

The coordinate system and the rotation axis of an *X*-ray imaging device had to be defined prior to defining the location of the delivery catheter. Figure 3a presents a 3D view during an endovascular intervention while using the MNS and C-arm, and the C-arm could change the shooting angle by rotating about the Y-axis (red line) as much as oblique angle α. Figure 3b shows the oblique degree α of the C-arm on the XZ plane, and α is defined as positive in the counterclockwise direction and negative in the clockwise direction. In this section, we present a mathematical algorithm that calculates the position information of the delivery catheter in the cross-section of the blood vessel by using two images taken at different oblique angles under the condition that the rotational axis of the C-arm coincides with the axial direction of the blood vessel.

**Figure 4:**
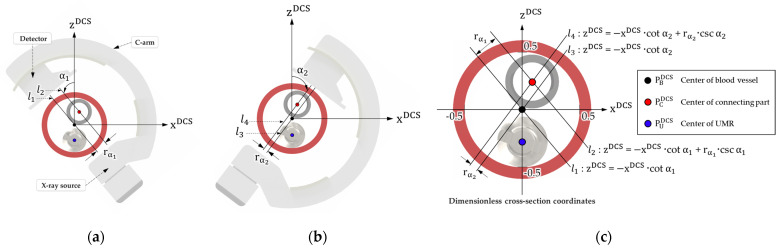
(**a**) Representation of r_α1_ in the cross-section of the blood vessel when the oblique angle is α_1_; (**b**) representation of r_α2_ in the cross-section of the blood vessel when the oblique angle is α_2_; (**c**) DCSs with the vessel center $P_{B}^{DCS}$, the center of the delivery catheter’s connecting part $P_{C}^{DCS}$, and the UMR’s center $P_{U}^{DCS}$.

Figure 3c shows a vascular image in which the delivery catheter and UMR are located and the axial direction of the blood vessel coincides with the Y-axis. The central axis of the blood vessel and delivery catheter in the image appears as two parallel lines along the Y-axis. Since the image appears differently depending on the oblique angle of the C-arm and the distance between the delivery catheter and the *X*-ray source, a pixel coordinate system was introduced to express the relative distance between the objects in the image. In Figure 3c, the pixel point $P_{N}^{pixel}$ in the image is a two-dimensional coordinate ($x_{N}^{pixel}$, $y_{N}^{pixel}$). $P_{1}^{pixel}$, $P_{2}^{pixel}$, and $P_{4}^{pixel}$ represent the leftmost, central, and rightmost pixel points of the cross-section of the blood vessel, and $P_{3}^{pixel}$ represents the central pixel point of the delivery catheter. Since the cross-section of the blood vessel is assumed to be circular and the axial direction of the blood vessel is assumed to coincide with the rotational axis of the C-arm, $x_{4}^{pixel}-x_{1}^{pixel}$ of the blood vessel in the image are equal to the diameter of the blood vessel, regardless of the shooting angle. By utilizing these characteristics, the distance ${\bar{P_{2}P_{3}}}^{pixel}$ to be measured is transformed into the ratio r_α_ with respect to the vessel diameter ${\bar{P_{1}P_{4}}}^{pixel}$, as shown in the following equation:

(3) $$r_{\alpha }=\frac{x_{3}^{pixel}-x_{2}^{pixel}}{x_{4}^{pixel}-x_{1}^{pixel}}$$

where r_α_ depends on the oblique degree α, which is defined as a negative value when the delivery catheter’s central axis is located on the left side of the center of the blood vessel and as a positive value when it is on the right side. This study proposes the dimensionless cross-sectional coordinates (DCSs), which define the inner diameter of the blood vessel as 1, as shown in Figure 4. DCSs are a two-dimensional dimensionless coordinate system with the blood vessel’s center $P_{B}^{DCS}$ as the origin.

Figure 4 shows the location of $P_{C}^{DCS}$, the center of the connecting part of the delivery catheter, in the cross-section of the blood vessel while using r_α1_ and r_α2_ in the DCSs. *l*_N_ in Figure 4 shows the line of the *X*-ray from the *X*-ray source to the detector of the *X*-ray as a straight line on the XZ plane. *l*_1_ and *l*_2_ in Figure 4a are straight lines that pass through $P_{B}^{DCS}$ and $P_{C}^{DCS}$ when the oblique angle of the *X*-ray C-arm is α_1_. *l*_3_ and *l*_4_ in Figure 4b are straight lines passing through $P_{B}^{DCS}$ and $P_{C}^{DCS}$ when the oblique angle is α_2_. r_α1_ and r_α2_ are the distances between parallel straight lines *l*_1_ and *l*_2_ and *l*_3_ and *l*_4_, respectively. $P_{C}^{DCS}$ can be expressed with Equation (4) in the DCSs by calculating the intersection of *l*_2_ and *l*_4_ in Figure 4c.

(4) $$P_{C}^{DCS}=\left(\frac{r_{\alpha_{1}}\cdot \sin\alpha_{2}-r_{\alpha_{2}}\cdot \sin\alpha_{1}}{\sin\alpha_{1}\cdot \sin\alpha_{2}\cdot \left(\cot\alpha_{1}-\cot\alpha_{2}\right)},\frac{r_{\alpha_{2}}\cdot \cot\alpha_{1}\cdot \sin\alpha_{1}-r_{\alpha_{1}}\cdot \cot\alpha_{2}\cdot \sin\alpha_{2}}{\sin\alpha_{1}\cdot \sin\alpha_{2}\cdot \left(\cot\alpha_{1}-\cot\alpha_{2}\right)}\right)$$

The DCSs are described as a relative length based on the inner diameter of the blood vessel. Consequently, after extracting the inner diameter of the blood vessel through *X*-ray image segmentation [23], the DCS value ($x^{DCS}$, $z^{DCS}$) is multiplied by the inner diameter of the blood vessel to determine the center of the connecting part of the delivery catheter.

### 2.3. Magnetic Field Control for Recombining the UMR with the Delivery Catheter

#### 2.3.1. Magnetic Force and Rotating Magnetic Field Applied to the UMR

To align the central axis of the UMR with the central axis of the connecting part of the delivery catheter, the UMR must move to the connecting part of the delivery catheter after compensating for the gravity of the UMR. After the UMR is moved to the connecting part of the delivery catheter, rotational motion of the UMR must be generated to connect the male screw of the UMR to the female screw of the connecting part of the delivery catheter.

**Figure 5:**
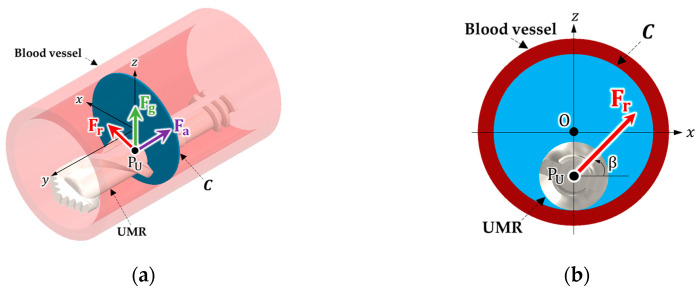
(**a**) Magnetic forces applied to the UMR (**F_g_**, **F_r_**, and **F_a_**); (**b**) magnetic force **F_r_** applied to the radial direction of the blood vessel and the angle β between **F_r_** and the X-axis of the blood vessel.

Figure 5a shows the magnetic forces, **F_g_**, **F_r_**, and **F_a_** that are to be applied to the UMR in order to be connected with the connecting part of the delivery catheter. **F_g_**, **F_r_**, and **F_a_** represent the magnetic force for gravity compensation for the UMR, the magnetic force in the radial direction of the blood vessel, and the magnetic force in the axial direction of the blood vessel, respectively. In this paper, magnetic force is generated with the assumption that the axial direction of the UMR and delivery catheter coincides with the Y-axis, and the cross-section of the blood vessel ***C*** is on the XZ-plane. The magnetic force **F_g_** for compensating for the UMR’s gravity is the multiplication of the UMR’s mass and the gravitational acceleration, and it acts along the +Z-axis direction. By applying a magnetic force **F_a_** in order to recombine the UMR, the UMR can reach the connecting part of the delivery catheter in the axial direction of the blood vessel. As shown in Figure 5a, **F_a_** must be applied to the negative direction of the central axis of the UMR as follows:

(5) $$F_{a}=-F_{axial}j$$

where *F_axial_* is the magnitude of **F_a_**.

As shown in Figure 5b, **F_r_** exists in the cross-section of the blood vessel and can be expressed with the following equation:

(6) $$F_{r}=F_{radial}(\cos\beta i+\sin\beta k)$$

where *F_radial_* is the magnitude of **F_r_** and β is the angle defined by using the X-axis.

**Figure 6:**
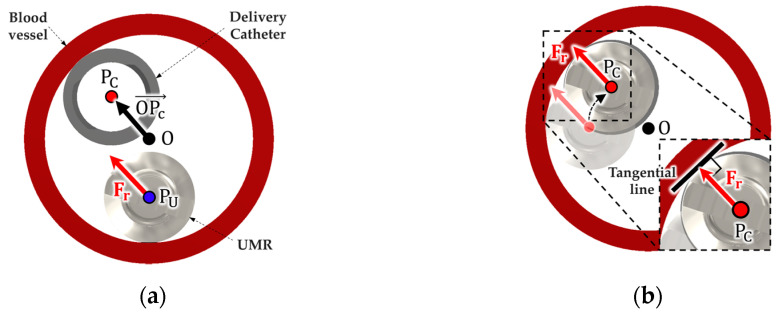
(**a**) **F_r_** in the direction from the center of the blood vessel to the center of the delivery catheter in the cross-section of the blood vessel; (**b**) a UMR moving to the point where the direction of **F_r_** makes a right angle with respect to the tangential vector of the inner wall of the blood vessel.

**Figure 7:**
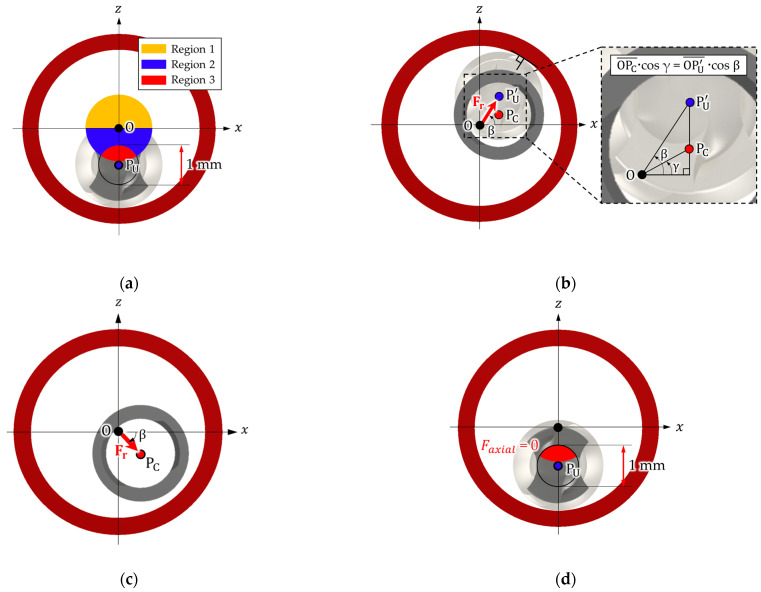
(**a**) Possible region of the center of the connecting part of the delivery catheter; (**b**) direction of **F_r_** and β in region 1; (**c**) direction of **F_r_** and β in region 2; (**d**) **F_r_** in region 3.

Figure 6 shows the case where the connecting part of the delivery catheter is located at the inner wall of the blood vessel. In Figure 6, O is the center of the vessel, P_C_ is the center of the connecting part of the delivery catheter, and $\vec{OP_{C}}$ is a vector directed from O to P_C_ in the cross-section of the blood vessel. Figure 6a shows that **F_r_** is applied to the UMR in the direction parallel to $\vec{OP_{C}}$. As shown in Figure 6b, the UMR moves to the point where the direction of **F_r_** makes a right angle with respect to the tangential vector of the inner wall of the blood vessel. However, the connecting part of the delivery catheter can be randomly located within the blood vessel. Figure 7 shows how to set the magnetic force **F_r_** according to the location of the delivery catheter to easily combine the UMR with the delivery catheter. In Figure 7, P_U_ is the center of the UMR in the cross-section of a blood vessel.

Figure 7a shows a possible region of the center of the connecting part of the delivery catheter. Region 1 in Figure 7a is a region in which the Z coordinate of P_C_ is positive, and Figure 7b shows the direction of **F_r_** in region 1. The direction of **F_r_** in region 1, including the gravity of the UMR, is toward the point P_U_^′^, and the direction β of **F_r_** in region 1 is determined by $\vec{OP_{C}}\;cos\gamma =\;\vec{O{P^{\prime }}_{U}}\;cos\beta$. After applying magnetic force in order to move the UMR to P_U_^′^ and terminating the magnetic force, the UMR moves in the −Z direction through a point coincident with P_C_ due to gravity. Figure 7c represents the direction of **F_r_** when the center of the connecting part of the delivery catheter is in region 2 and the Z-coordinate of P_C_ is negative. In region 3, as shown in Figure 7d, **F_r_** is not applied because the center of the UMR is very close to the center of the connecting part of the delivery catheter (less than 1 mm). After all, the magnetic force **F_m_**, required to align the UMR with the connecting part of the delivery catheter can be expressed with the following equation:

(7) $$F_{m}=F_{radial}\cos\beta i-F_{axial}j+(F_{radial}\sin\beta +m_{U}g)k$$

where *m_U_* is the mass of the UMR and *g* is the gravitational acceleration (9.8 m/s^2^).

**Figure 8:**
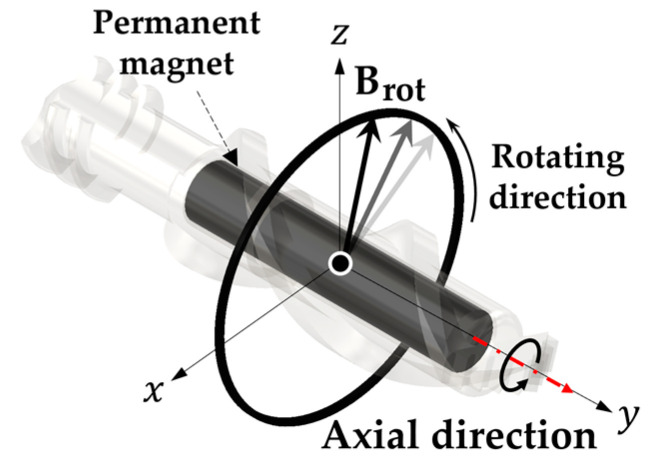
Rotating magnetic field applied to the UMR.

When the application of the magnetic force **F_m_** is terminated, the screw part of the UMR must be engaged with the connecting part of the delivery catheter through the rotational motion of the UMR. Figure 8 shows a rotational magnetic field **B_rot_** that generates rotational motion of the UMR for recombination. **B_rot_** is on the XZ-plane and is expressed with the following equation:

(8) $$B_{rot}(t)=B_{amp}(\cos2\pi fti-\sin2\pi ftk)$$

where *B_amp_* and *f* are the magnetic flux density and rotation frequency of the rotating magnetic field, respectively.

#### 2.3.2. Procedure for Recombining the UMR with the Delivery Catheter

**Figure 9:**
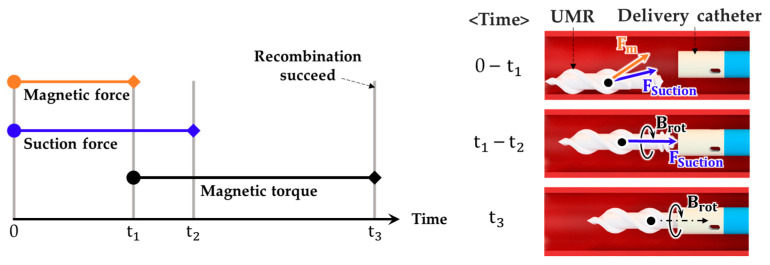
Procedure for recombining the UMR with the delivery catheter by applying magnetic force **F_m_**, a rotational magnetic field **B_rot_**, and suction force to the UMR.

We propose a process for recombining the UMR with the delivery catheter, as shown in Figure 9.

(1) Two images of the delivery catheter in the blood vessel with different oblique angles are obtained from the *X*-ray image device. The central position of the connecting part of the delivery catheter is determined with the algorithm proposed in Section 2.2.
(2) **F_m_** is applied during t_1_ to align the UMR with the delivery catheter. Simultaneously, the feeding robot performs suction through the delivery catheter to assist with **F_m_**. **F_m_** and suction force are used to align the UMR with the connecting part of the delivery catheter. The application of **F_m_** and the suction force starts simultaneously, and the application of **F_m_** is completed before completing the suction force.
(3) **B_rot_** is applied from t_1_, and it causes the rotational motion of the UMR in order to combine the UMR with the delivery catheter. The suction force finishes at t_2_. **B_rot_** is applied until recombination is complete at t_3_.
(4) Fluid of the delivery catheter is injected into the inner hole to check if the delivery catheter and UMR are separated.

## 3. Experiment and Verification

### 3.1. In Vitro Experiment

**Figure 10:**
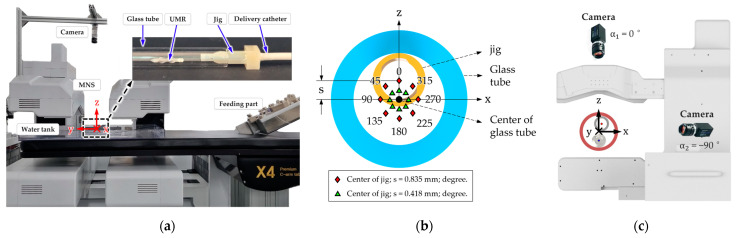
(**a**) Experimental setup for the in vitro experiment on the retrieval process; (**b**) 17 recombining positions; (**c**) oblique angle (α_1_ = 0°, α_2_ = −90°) and shooting direction of the RGB camera.

To verify the algorithm in Section 2.2 and the retrieval process in Section 2.3, an in vitro experiment was conducted in a glass tube to simulate a vascular environment, as shown in Figure 10a. The glass tube, which had an inner diameter of 4 mm, was placed inside a water tank, and a UMR was placed inside it. We designed a jig to fix the connecting part of the delivery catheter, which had a diameter of 2.33 mm. The jig fixed the connecting part (screw part) in one of 17 positions in the cross-section of the glass tube. Figure 10b shows the 17 positions within the region while considering the radii of the glass tube and connecting part, which were 2 mm and 1.17 mm, respectively. S was the distance from the center of the glass tube to the center of the connecting part. The center of the connecting part was located at the outermost part of the glass tube when the distance s was 0.835 mm. The 17 positions were set as recombining positions; they included the center of the glass tube and 16 points along the circumferential direction with the interval of 45°, where s was equal to 0.835 mm (outermost) and 0.418 mm (middle). The delivery catheter was connected to the opposite side of the jig. In addition, two RGB cameras were fixed on the Z- and X-axes of the MNS’s operating region. Figure 10c shows the shooting angles (α_1_ = 0°, α_2_ = −90°) in the XZ plane used to obtain two images. As shown in Figure 10a, the axial directions of the glass tube, delivery catheter, and UMR coincided along the +Y-axis.

**Figure 11:**
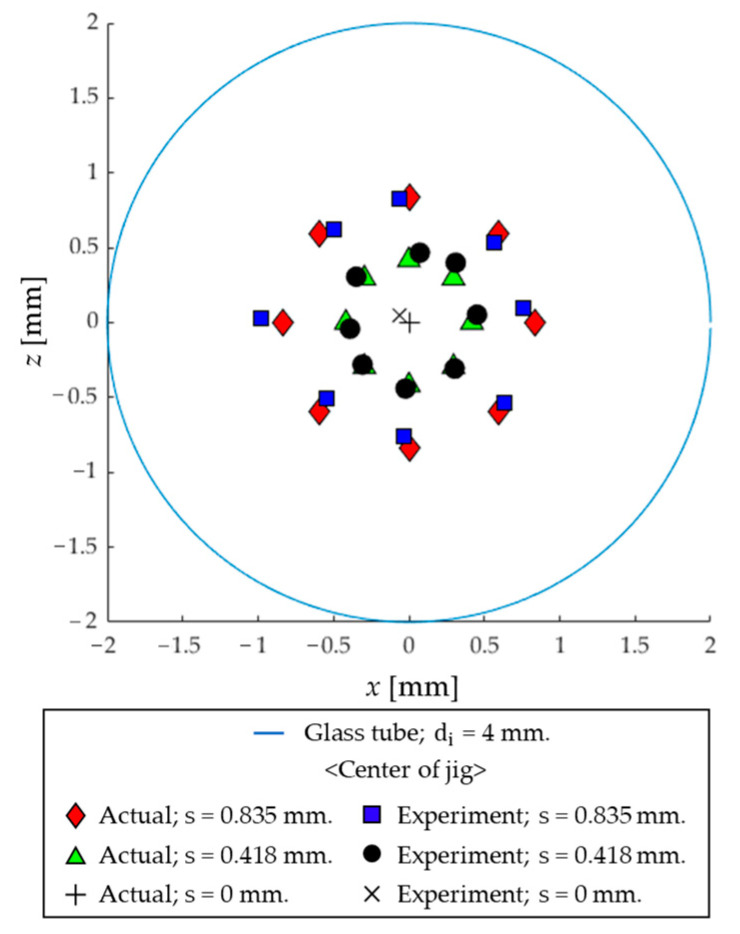
Actual and calculated positions on the cross-section of the glass tube (XZ-plane).

The algorithm presented in Section 2.2 was utilized to determine the location of the connecting part in the cross-section of the glass tube by using the images that were shot. Table 1 and Figure 11 show the results of a comparison of the actual and calculated positions for 17 points at the center of the connecting part. The calculated positions matched within error ranges of 7.41% and 8.31% for the X- and Z-coordinates, respectively, with respect to the actual positions, and it was confirmed that the average error of the X and Z coordinates was within 0.05 mm. The proposed algorithm could be used to determine the position of the connecting part of the delivery catheter.

**Figure 12:**
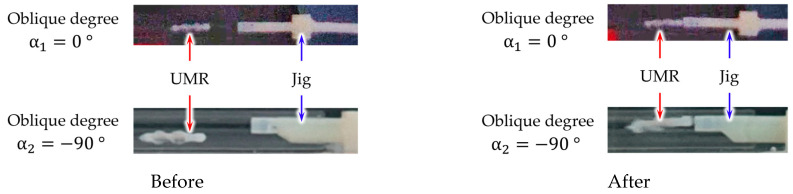
Images of the UMR and the delivery catheter in the jig aligned in the glass tube before and after applying magnetic force and suction force (camera direction: α_1_ = 0°, α_2_ = −90°).

After recognizing the position of the connecting part, the retrieval process of the UMR was performed in the glass tube to experimentally verify its effectiveness. The magnitudes of **F_r_**, **F_a_**, and **F_g_** were set to 0.2 mN, 0.2 mN, and 0.39 mN, respectively. Simultaneously, a suction force of 74 mN (flow rate of 3.6 mL/s) was also applied. As shown in Figure 12, the axes of the UMR and connecting part of the jig were aligned in the glass tube through the retrieval process proposed in Section 2.3.2. Then, a rotational magnetic field **B_rot_** with a magnetic flux density of 10 mT and a rotation frequency of 10 Hz was generated to combine the UMR with the connecting part of the delivery catheter. In the retrieval experiment, the times taken to apply the magnetic force **F_m_** and the suction force were 2 s (t_1_) and 3 s (t_2_), respectively.

**Figure 13:**
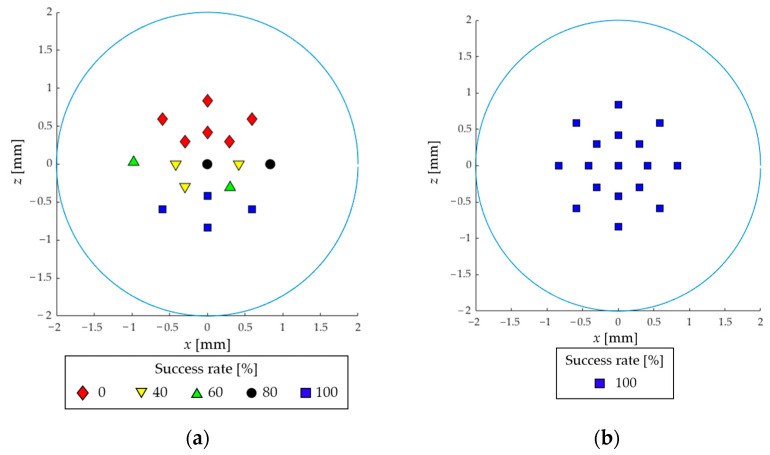
(**a**) Success rate of recombination of the UMR with the delivery catheter without applying magnetic force; (**b**) with applying magnetic force.

Figure 13a shows the success rate of a retrieval process that was applied only with a rotating magnetic field and suction force and without applying magnetic force. In this case, a magnetic force used to lift the UMR could not be generated when the connecting part was located at the top of the glass tube. The UMR stayed at the bottom of the glass tube and was not engaged with the connecting part of the delivery catheter, and the retrieval success rate decreased. When s was 0.835 mm and 0.418 mm in the 0°, 45°, and 315° directions, the UMR hovered around the outside of the connecting part or remained stuck between the inner wall of the glass tube and the connecting part. When s was 0.418 mm in the 90°, 135°, 225°, and 270° directions, the retrieval success rate was decreased because the UMR was not aligned with the connecting part of the delivery catheter when only suction force was used. Figure 13b shows the retrieval success rate of the UMR when utilizing the proposed retrieval process. The retrieval success rate was significantly improved to 100% in all 17 possible locations.

### 3.2. In Vivo Experiment

The retrieval process of the UMR during an endovascular intervention by using the I-RAMAN system and an SRMR was verified in the femoral arteries of two male Jeju pigs with an age of 6 months. The diameters of the left femoral arteries of the pigs were 3 mm (1) and 3.57 mm (2), and those of the right femoral arteries were 3.5 mm (1) and 3.64 mm (2). This experiment was conducted under the Animal Protection Act (Law No. 16544, enforced on 27 August 2019) and the Laboratory Animal Act (Law No. 15944, enforced on 12 March 2019), and it was approved by the Animal Experimental Ethics Committee in Cardiocerebrovascular Disease Efficacy Evaluation Support Center of Yonsei University (Animal Test Approval Number: CPEC-IACUC-22-1008).

After placing the femoral artery of a pig inside the operating region of the MNS, a commercial guiding sheath (diameter of 7 Fr) was inserted through the femoral artery near the lesion, and an SRMR was inserted through the guiding sheath. Then, a contrast agent was injected through the delivery catheter, and *X*-ray images were obtained by using the C-arm with oblique angles of 0° and 15° for the left femoral artery and angles of 0° and −15° for the right femoral artery in order to create a three-dimensional vascular path that contained information on the position and direction of the blood vessel [24].

**Figure 14:**
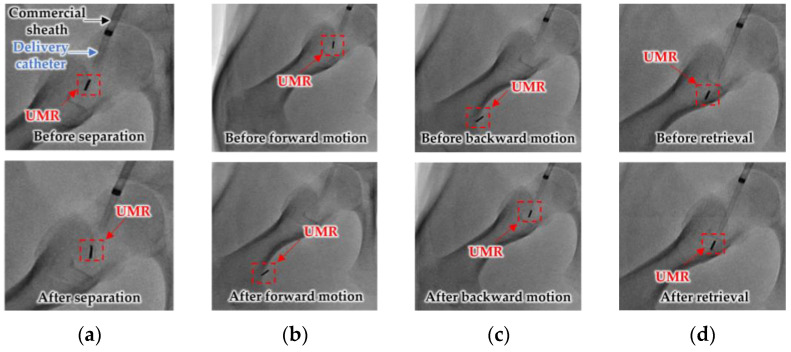
*X*-ray images from the in vivo experiment: (**a**) separation; (**b**) forward motion; (**c**) backward motion; (**d**) retrieval.

Figure 14 shows the experimental procedure using the I-RAMAN system and SRMR after the SRMR was inserted into the blood vessel near the lesion. In Figure 14a, the UMR was separated from the delivery catheter by applying a rotating magnetic field with a magnetic flux density of 10 mT and a frequency of 10 Hz in the direction of the blood vessel. In Figure 14b, a rotating magnetic field with a magnetic flux density of 15 mT and a frequency of 16 Hz was applied to cause a forward motion of the UMR toward the lesion. Afterward, as shown in Figure 14c, under the same conditions, the rotating magnetic field used for the forward motion of the UMR was applied in the opposite direction to cause a backward motion of the UMR in order to move it into the vicinity of the connecting part. As shown in Figure 14d, by using the procedure proposed in Section 2.3.2, the delivery catheter and UMR were successfully recombined. In this instance, the magnitude and duration of the magnetic force and suction force were set to the same values as those in the in vitro experiment. Table 2 shows the center of the connecting part of the delivery catheter as calculated by using the algorithm presented in Section 2.2, as well as the success rate of the retrieval of the UMR by using the algorithm presented in Section 2.3. This showed that the retrieval of the UMR was 100% successful for four sites in the right and left femoral arteries of the two pigs. After the retrieval procedure, a contrast agent was injected through the hole of the delivery catheter, and we confirmed that the UMR had not separated.

## 4. Conclusions

We proposed a method of identifying the position of a tethered delivery catheter in a vascular environment, recombining a UMR with a tethered delivery catheter, and safely retrieving them from a vascular environment in an endovascular intervention by utilizing an SRMR and MNS. From images of a blood vessel and tethered delivery catheter taken from two different angles, we developed a method of extracting the position of the delivery catheter. Then, we also developed a retrieval method for the UMR by using the magnetic force while considering the delivery catheter’s position, suction force, and a rotating magnetic field. In the in vitro experiment, which was performed in a glass tube environment with an RGB camera, we confirmed that the location of the delivery catheter in the glass tube could be recognized with an average error of 0.05 mm in each of the X- and Z-coordinates and that the retrieval success rate was greatly improved in comparison with the case without the use of magnetic force. In an in vivo experiment, we successfully retrieved the UMR from the femoral arteries of pigs. This research will contribute to the realization of robotic endovascular interventions.

## Figures and Tables

**Table 1:** Actual and calculated positions of the 17 points that were used as the center of the connecting part.

S [mm]	Position ^1^ [°]	X (Cal.) ^2^ [mm]	Z (Cal.) ^2^ [mm]	X (Actual) [mm]	Z (Actual) [mm]	Error (X) [mm]	Error (Z) [mm]
0.835	0	−0.06	0.83	0.00	0.84	0.06	0.01
	45	−0.50	0.63	−0.59	0.59	0.09	0.04
	90	−0.98	0.03	−0.84	0.00	0.14	0.03
	135	−0.55	−0.51	−0.59	−0.59	0.04	0.08
	180	−0.03	−0.76	0.00	−0.84	0.03	0.08
	225	0.63	−0.53	0.59	−0.59	0.04	0.06
	270	0.76	0.09	0.84	0.00	0.08	0.09
	315	0.57	0.53	0.59	0.59	0.02	0.06
	0	0.07	0.47	0.00	0.42	0.07	0.05
0.418	45	−0.35	0.31	−0.30	0.30	0.05	0.01
	90	−0.39	−0.04	−0.42	0.00	0.03	0.04
	135	−0.31	−0.28	−0.30	−0.30	0.01	0.02
	180	−0.02	−0.44	0.00	−0.42	0.02	0.02
	225	0.30	−0.31	0.30	−0.30	0.01	0.01
	270	0.45	0.05	0.42	0.00	0.03	0.05
	315	0.31	0.40	0.30	0.30	0.01	0.10
0	center	−0.06	0.05	0.00	0.00	0.06	0.05
Average error [mm]						0.05	0.05

^1^ Angles formed by the center of the connecting part with respect to the Z-axis. ^2^ Positions calculated by using the algorithm presented in Section 2.2.

**Table 2:** Calculated central position of the connecting part of the delivery catheter and the success rate of the retrieval of the UMR from the femoral arteries of the pigs.

Pig *	Leg	Oblique Degree		P_C_ (x, z) [mm]	Retrieval Result
		α_1_ [°]	α_2_ [°]		
1 *	Right	0	−15	(0.11, −1.29)	Success
	Left	0	+15	(−0.25, −0.35)	Success
2 *	Right	0	−15	(−0.6, 1.13)	Success
	Left	0	+15	(0.6, 0.16)	Success

* Pigs 1 and 2 underwent the retrieval process at a total of four sites, with two sites (right leg, left leg) each.

## Data Availability

Not applicable.
